# High Data Rate Communications In Vivo Using Ultrasound

**DOI:** 10.1109/TBME.2021.3070477

**Published:** 2021-10-19

**Authors:** Zhengchang Kou, Rita J. Miller, Andrew C. Singer, Michael L. Oelze

**Affiliations:** Electrical and Computer Engineering Department, University of Illinois, Urbana-Champaign, USA.; Beckman Institute, University of Illinois, USA.; Electrical and Computer Engineering Department, University of Illinois, Urbana-Champaign, USA.; Electrical and Computer Engineering Department, University of Illinois, Urbana-Champaign, Urbana, IL 61801 USA

**Keywords:** Diversity receiver, implanted medical device, in-body communications, OFDM modulation, wireless communication

## Abstract

The emergence of in-body medical devices has provided a means of capturing physiological or diagnostic information and streaming this information outside of the body. Currently, electromagnetic-based communications make up the bulk of in-body medical device communication protocols. Traditional electromagnetic-based solutions are limited in their data rates and available power. Recently, ultrasound was investigated as a communication channel for through-tissue data transmission. To achieve real-time video streaming through tissue, data rates of ultrasound need to exceed 1 Mbps. In a previous study, we demonstrated ultrasound communications with data rates greater than 30 Mbps with two focused ultrasound transducers using a large footprint laboratory system through slabs of lossy tissues. While the form factor of the transmitter is also crucial, it is obvious that a large, focused transducer cannot fit within the size of a small in-body medical device. Several other challenges for achieving high-speed ultrasonic communication through tissue include strong reflections leading to multipath effects and attenuation. In this work, we demonstrate ultrasonic video communications using a mm-scale microcrystal transmitter with video streaming supplied by a camera connected to a Field Programmable Gate Array (FPGA). The signals were transmitted through a tissue-mimicking phantom and through the abdomen of a rabbit *in vivo*. The ultrasound signal was recorded by an array probe connected to a Verasonics Vantage system and decoded back to video. To improve the received signal quality, we combined the signal from multiple channels of the array probe. Orthogonal frequency division multiplexing (OFDM) modulation was used to reduce the receiver complexity under a strong multipath environment.

## I. Introduction

THE GROWTH of in-body or implanted medical devices has accelerated over the past decades. As the amount of information collected by these devices increases, the need for developing communication protocols to wirelessly, rapidly and safely transmit this information outside the body has also increased. In some in-body devices, there is a need to transmit video from inside the body to outside the body.

One such device that requires data rates sufficient to transmit video is capsule endoscopy [2]-[6]. Capsule endoscopy is employed clinically to provide diagnostic information for diseases of the stomach and small intestines. This approach is less invasive and is, therefore, preferred, when possible, over traditional methods where a scope on a cable is traversed down the esophagus and into the stomach and small intestines. Capsule endoscopy systems, i.e., camera pills, have been created to wirelessly transmit image data through the human body as the pill traverses through the small intestines. Currently, all wireless camera pills transmit data via an electromagnetic (EM) communication modality to a receiving antenna outside the body, where the signal is decoded, and images stored for later viewing.

Traditional EM-based wireless communication approaches face several limitations for in-body communication, especially in the hospital environment. First, the high attenuation of EM waves inside the human body can result in low signal-to-noise ratio (SNR) and limited penetration depth. Second, the power supply of small in-body devices is limited because the batteries must be small to fit in the devices, i.e., the battery capacity is limited to less than 100 mAh. To keep working for more than 8 hours, the total power is limited to less than 40 mW, which limits the transmit power. Third, the temperature rise caused by the absorption of EM energy by tissues also limits the transmit power. Because of these limitations, in-body medical devices using EM wave communication in the human body have not achieved data rates capable of real-time video streaming.

To address some of these issues, in one study [7] a conformal helical antenna was investigated as a transmitter antenna to reduce the elevation in temperature resulting from EM radiation. The authors demonstrated a data rate of 256 kbps with a low error rate (<10^−3^) in an *in vivo* test with specific absorption rate well below FCC recommendation. In other work [8], a backscatter communication approach was used to move all the communication power consumption outside of the body. In that study, a data rate of up to 1 Mbps was achieved over an 8-cm distance at 0 dBm transmitting power. However, the system occupied the 25 MHz RF bandwidth, which has the potential to interfere with other equipment using similar bands and violates current FCC regulations.

As an alternative to EM communications, ultrasound can provide a communication channel in the human body that has the potential to provide sufficiently high data rates to stream real-time high-definition (HD) video, which requires 5 Mbps data rate for 1280*720 resolution at 30 frame per second. Because it is ultrasound, it will not interfere with other equipment in the hospital setting. To achieve over 5 Mbps reliable data transmission, high uncoded data rates are needed to compensate for the cost of channel coding. The modulation order is also limited by the signal-to-noise ratio (SNR). Considering these limitations, a bandwidth of more than 1 MHz is needed. Furthermore, to increase penetration depth, a low carrier frequency is required to alleviate the attenuation caused by tissue. This represents an engineering tradeoff between frequency and bandwidth and penetration depth.

In a previous study, we demonstrated high-speed communication using ultrasound through tissue [1], with data rates of up to 30 Mbps achieved through 5-cm thick pork loin and beef liver samples. The transducer used in the study had a wide bandwidth (−10-dB bandwidth of 5 MHz), a center frequency of 4 MHz and large diameter (1.92 cm). In that system, the communication waveforms were generated by an arbitrary waveform generator connected to a power amplifier that was then connected to the transmitting transducer. The matched receiving transducer was connected to a computer for processing. This laboratory system was used to demonstrate the capability of ultrasound to transmit high data rates through tissues sufficient for streaming video. However, the footprint of the laboratory system required a small table to house it and, therefore, would not be small enough to fit inside a small in-body device.

To enable in-body ultrasound communications with a footprint small enough to fit in a small in-body device, like an endoscopy capsule, and with data rates capable of streaming video requires small transducer elements, a small processing unit and sufficient ultrasonic power. In a recent study by Bos, et al., small individual ultrasonic elements, i.e., ultrasonic microcrystals (Sonometrics, London Ontario), were used as transmit and receive transducers [9], [10]. These microcrystals are 2 mm in diameter, operate at center frequency of 1.2 MHz, are biocompatible and have low directivity. In one of those studies, data rates of 4.4 Mbps were achieved through beef liver with quadrature amplitude modulation (QAM) and a decision feedback equalizer [10]. Bos et al. used QAM and orthogonal frequency division multiplexing (OFDM) modulation to achieve data rates over 300 kbps through beef samples and over distance of 10 cm [11]. These studies demonstrated that using ultrasound as a communication medium with OFDM was feasible for in-body communication with small footprint transducers. However, the data rates achieved were insufficient for real-time video transmission.

In studies by Santagati et al. [12], [13], an IoT network using ultrasound was implemented. In that work, they used a customized 9.5-mm diameter thin disk piezoelectric transducer and pulse-position modulation (PPM). The processing system consisted of a small footprint FPGA and microcontroller. A physical layer was implemented on an FPGA while a media access control layer was positioned on a microcontroller unit to maximize battery life. The use of PPM also simplified the transmission because there was no need to use an external DAC. The network they built was suitable for low speed data or control signal transmission. They achieved data rates of 180 kbps at −20-dBm transmit power. However, they did not provide video capable data rates using ultrasound with a small footprint processing unit.

In this study, we demonstrate 1) streaming of real-time video using ultrasound as a communication modality, 2) an ultrasonic array connected to a commercial ultrasonic scanner to capture the ultrasonic signals and 3) a small form factor source transmitting signals using 4) a small footprint FPGA board. The signals were received and processed using a commercial ultrasound scanner and array probe because there is less of a constraint for the size of the external receiving device. This work moves toward miniaturization of the transmission source for use in a small in-body medical device, like a capsule endoscopy, and how these signals might be captured for processing by an external array receiver. In this paper, Section II first describes the channel model we used to simulate the in-body communication environment and then describes both the methodology employed for the modulation and equalization schemes, the test setup and FPGA implementation of transmitter. Section III provides the results of the study for both the simulation and phantom-based test and images received in an *in vivo* test. The last section (Section IV) provides discussion and conclusions regarding the study.

## II. Methodology

### A. Ultrasound Channel Model

**Fig. 1.**
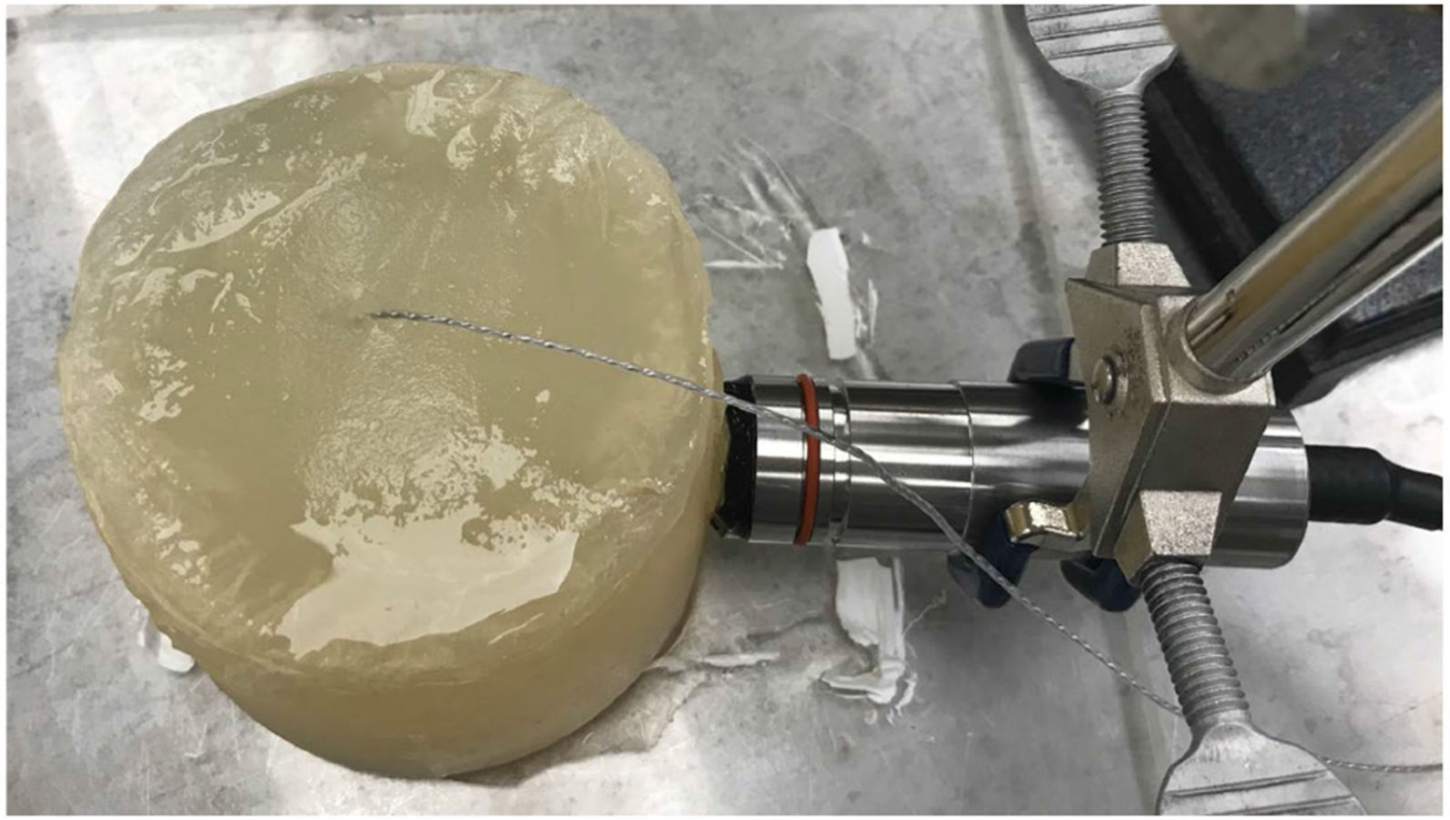
IP103 probe positioned along the side of the phantom. The cable attached to the microcrystal can be observed penetrating into the phantom at its center.

**Fig. 2.**
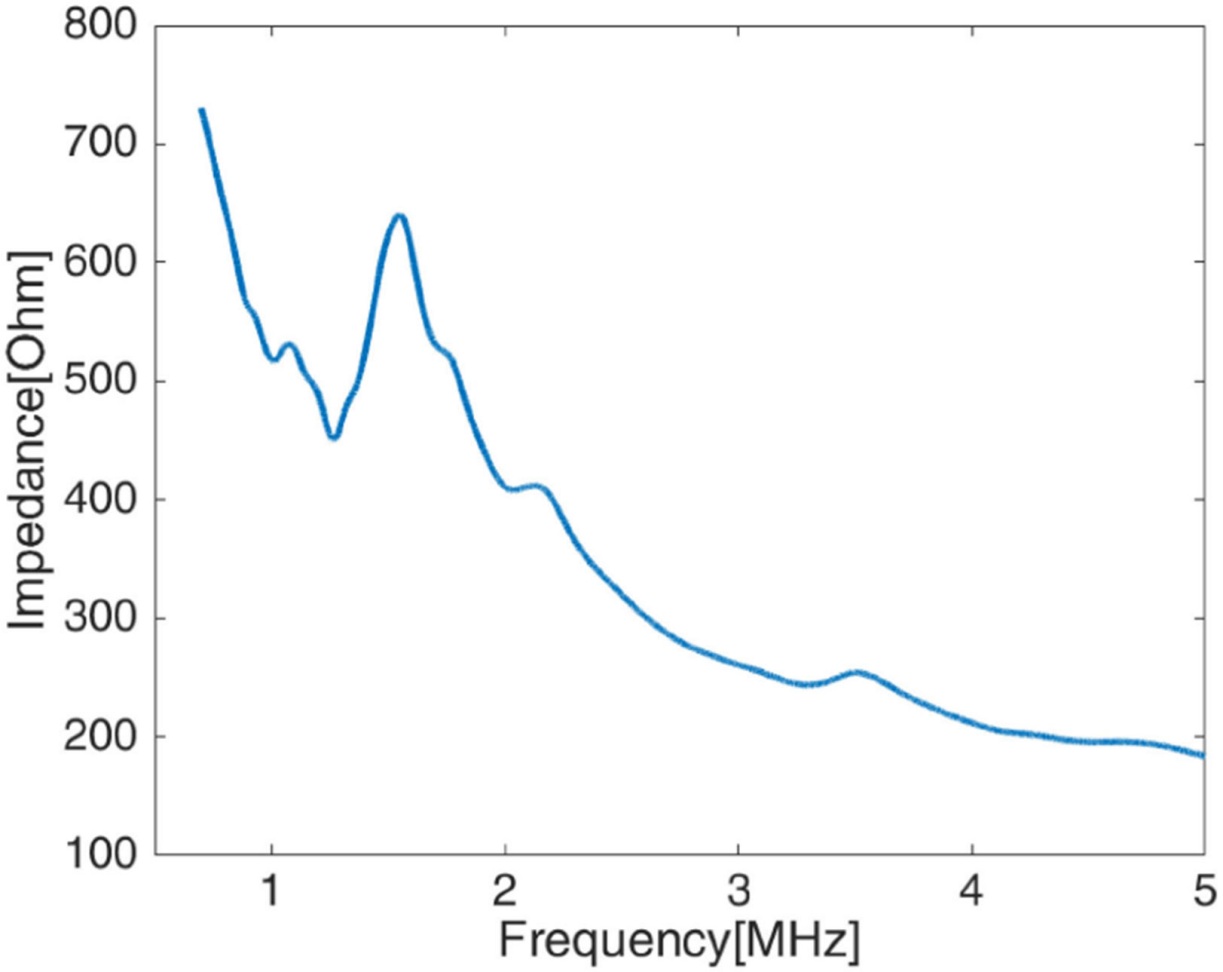
Impedance measurement of the microcrystal transducer.

To model and capture the characteristics of an in-body ultrasonic communication channel, i.e., to estimate what the delay spread due to multipath might be in a tissue region like the human abdomen, a small tissue-mimicking phantom with bones or other reflectors inside can be constructed and the finite-length impulse response (FIR) channel can be measured between transducers embedded inside the phantom and external receivers [9]. Here we constructed a cylindrical-shaped phantom to partially mimic the human abdomen. The phantom was 10 cm in diameter and 9 cm in height and was made with a 1.5% agar and 1.5% gelatin mixture as shown in Fig. 1. A microcrystal transducer (Sonometrics, London Ontario) was used as the transmitter. The microcrystal was unfocused, had a 2-mm diameter and a nominal center frequency of 1.2 MHz (measurements produced a center frequency of 1.4 MHz). The impedance measurement of the microcrystal is shown in Fig. 2. The microcrystal was embedded in the phantom 5 cm from the top and 6.5 cm from the edge of the phantom. In this experiment, an IP103 64-element phased array (Sonic Concepts, Bothell, WA) was used as the receiving array positioned along the side of the phantom. The array had a nominal center frequency of 3.2 MHz with an element height of 14 mm and pitch of 0.304 mm.

**Fig. 3.**
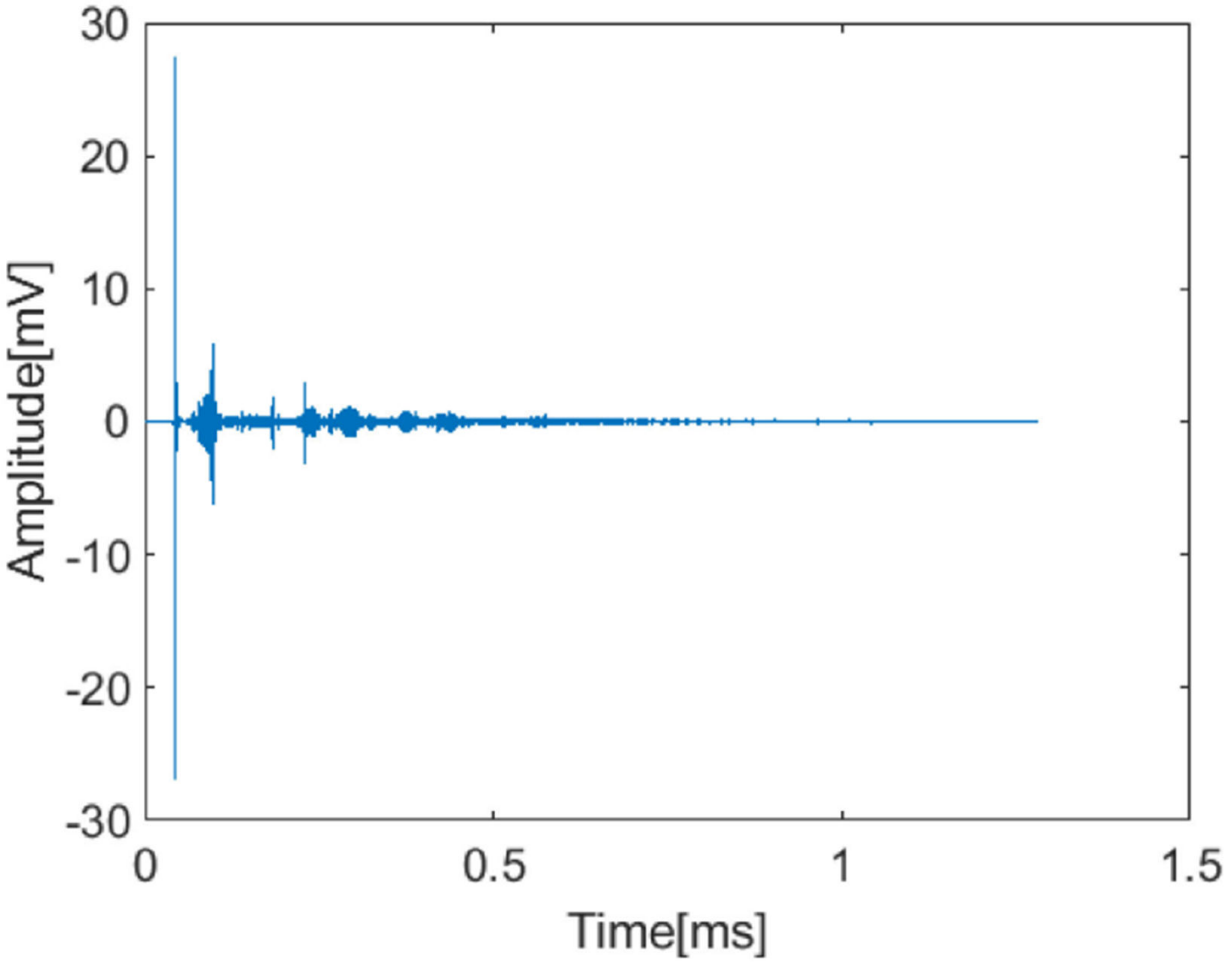
FIR channel measured through an abdominal phantom with the center element of receiving array. Delay spread was up to 1 ms long.

We estimated the FIR channel by generating a symbol sequence in Matlab (MathWorks, Natick, MA), loading the sequence into a Tabor WW1281 arbitrary waveform generator (AWG) (Tabor Electronics, Haifa, Israel), transmitting the sequence with the microcrystal and recording the received signal with the IP103 64-element phased array (Sonic Concepts, Bothell, WA) connected to a Verasonics Vantage 128 system (Verasonics, Kirkland, WA). The FIR channel measured by the center element of the probe is shown in Fig. 3. As the size of phantom was relatively large compared to a wavelength and strong reflections occurred at the boundary between the air and phantom material, delay spread up to 1 ms was observed. To minimize the performance loss due to the multipath delay spread, the symbol duration was chosen to be much longer than the delay spread, which results in a narrow subcarrier bandwidth. The frequency response of the channel was estimated over the occupied ultrasonic frequency band measured by all 64 elements.

### B. Modulation and Equalization Scheme

**Fig. 4.**
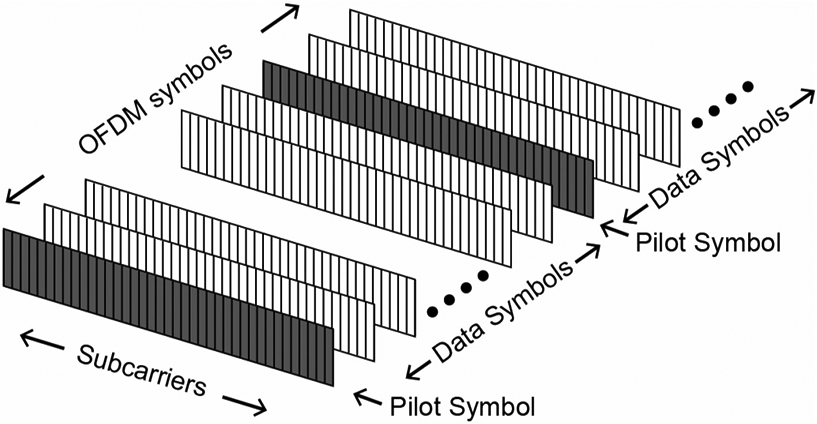
Frame structure of OFDM modulation used in this study. Shaded are pilot symbols, unshaded symbols between pilot symbols are data symbols.

According to the channel model measured from the phantom, it was observed that the delay spread was up to 1 ms, which results in a narrow coherence bandwidth. To mitigate this, we used OFDM modulation with a narrow subcarrier bandwidth to simplify the equalization process. QAM signaling was used in each subcarrier. The proposed OFDM frame structure was composed of pilot symbols and data symbols. Fig. 4 shows the details of the frame structure, also called a block type pilot [14].

The number of data symbols between two pilot symbols was determined by the rate of channel variation. For ultrasound in-body communication, the channel variation rate is much lower than the inverse of the time interval between two pilot symbols. In this design, we chose to set the interval to 12 data symbols. We used two pilot symbols to estimate the channel and interpolated through these data symbols to perform equalization. A pseudo noise (PN) sequence was used as the pilot signal. A block-type pilot sequence provided a higher channel estimation accuracy when the coherence bandwidth was narrow, and the channel variation rate was low compared to the distance between two pilot symbols. This is because we had the channel measurement at each individual subcarrier, compared to previous studies, which had pilot tones distributed amongst the subcarriers [11], [15].

To improve the SNR, the receiving area was increased by using an ultrasonic array as the receive transducer. Passive receive focusing was used to sum the signals from each channel to further boost the SNR. Compared to single-element focused transducers we could automatically focus on the transmit element with the array, reducing the requirement that the transmitter and receiver be perfectly aligned.

**Fig. 5.**
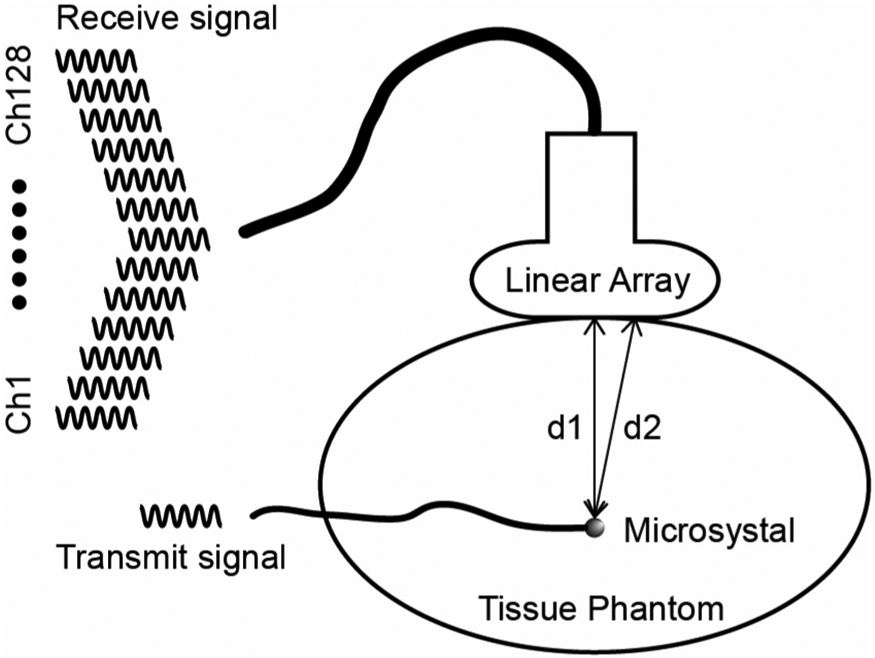
Illustration of time delay difference between receive signals from different elements of the linear array.

As Fig. 5 illustrates, there are path differences between the transmit microcrystal to different receive elements on the array, which results in different time delays to each of the receive channels. To passively focus on the transmit microcrystal we performed symbol timing recovery (STR) on each receive channel, which aligned all receive channels to the same time point up to the precision of sampling rate. The phase offsets caused by residual delay differences between channels were compensated by performing channel equalization on each channel’s subcarrier.

**Fig. 6.**
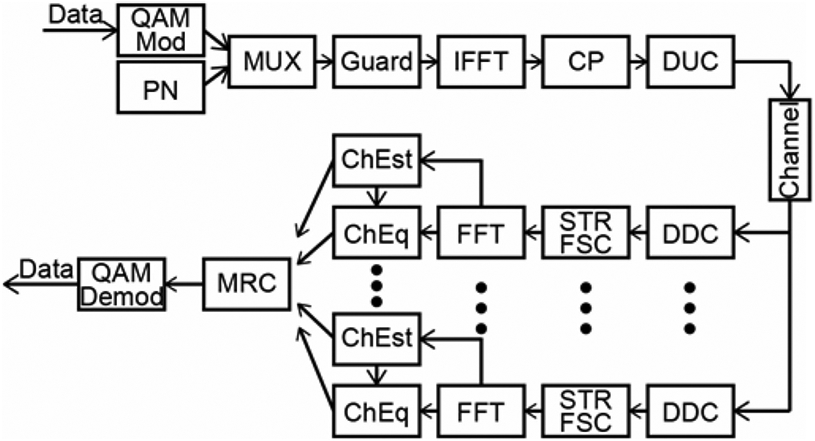
Structure of OFDM modulation and demodulation used in this study.

The overall processing chain is displayed in Fig. 6. On each of the multiple receive channels the following operations were performed: digital down conversion (DDC), STR, Frequency shift compensation (FSC), Fast Fourier transform (FFT), Channel Estimation and Equalization (ChEst, ChEq) individually. At the final stage, the product of all channels was combined together by maximum ratio combining (MRC) [16].

To mitigate inter symbol interference, a cyclic prefix (CP) based symbol timing recovery was used with the frequency shift estimation method [17]. After applying frequency offset estimation, we can correct for frequency offset and the CP symbol timing recovery provides the starting point of each OFDM symbol, from which we can set the start point of the FFT to perform the OFDM demodulation. Because the pilot symbol is obtained at the beginning and ending of one frame, which provides the pilot signal at each subcarrier, we can estimate the channel by dividing the received pilot signal by the transmitted pilot signal [18]. The channel estimate is interpolated through the data symbols between the two pilot symbols.

The anticipated multipath environment in a medium like the human abdomen causes frequency selective fading, which results in poor SNR for some subcarriers. To alleviate this problem, we applied a weighted combination of a subcarrier from different receive channels with weights selected according to the channel response magnitude for the specific subcarrier, i.e., MRC, if the frequency selective fading does not impact all channels.

**Fig. 7.**
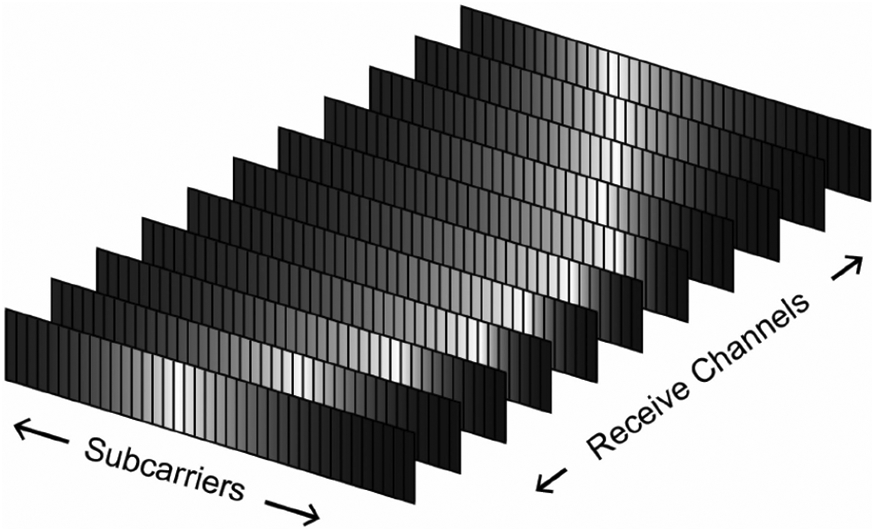
Illustration of different channel response magnitudes in different channels and subcarriers, where the brightness reflects how deep the frequency selective fading is.

As Fig. 7 illustrates, the brighter subcarrier corresponds to a stronger frequency selective fading. Because the receive elements are spatially distributed, we can have different fading profiles in different channels. Here we define the number of channels as *N*, the number of subcarriers as *B*, the equalized signal at subcarrier *i* and channel *j* is *S_i,j_*, *i* ∈ [1, *B*] *j* ∈ [1, *N*] and the magnitude of the channel response at the same position is *H_i,j_*, *i* ∈ [1, *B*] *j* ∈ [1, *N*]. Then, the MRC can be calculated as,

(1) $$C_{i}=\frac{\sum_{j=1}^{N}S_{i,j}*H_{i,j}}{\sum_{j=1}^{N}H_{i,j}}.$$

After calculating and applying the MRC product, the processed signal is quantized to the QAM symbol map and then demodulated to recover the original transmitted data.

The modulation parameters used in this study are listed in Table I. A 4096-point FFT was chosen, as compared to [11], which used 64 and 32768, respectively. A 64-point FFT was too small for our purpose as the subcarrier bandwidth would be too wide, while 32768 was too large to implement on a resource limited FPGA. The total bandwidth used was 937.5 kHz. With 16 QAM modulation, the overhead of CP and pilots, the payload data rate was 3 Mbps, which is sufficient for HD video streaming using H.264 encoding. With 256 QAM modulation, a higher data rate of 6 Mbps was achievable. We also applied the same modulation scheme with a higher baseband sampling rate and center frequency to use a larger bandwidth, which enabled even higher data rates of 15.2 Mbps. The higher bandwidth of 2.3 MHz was more than twice the original bandwidth. In this study, we did not include any channel coding or video coding because the aim of the study was to demonstrate the capability of using ultrasound as a channel for video capable capsule endoscopy.

### C. Test Setup

**Fig. 8.**
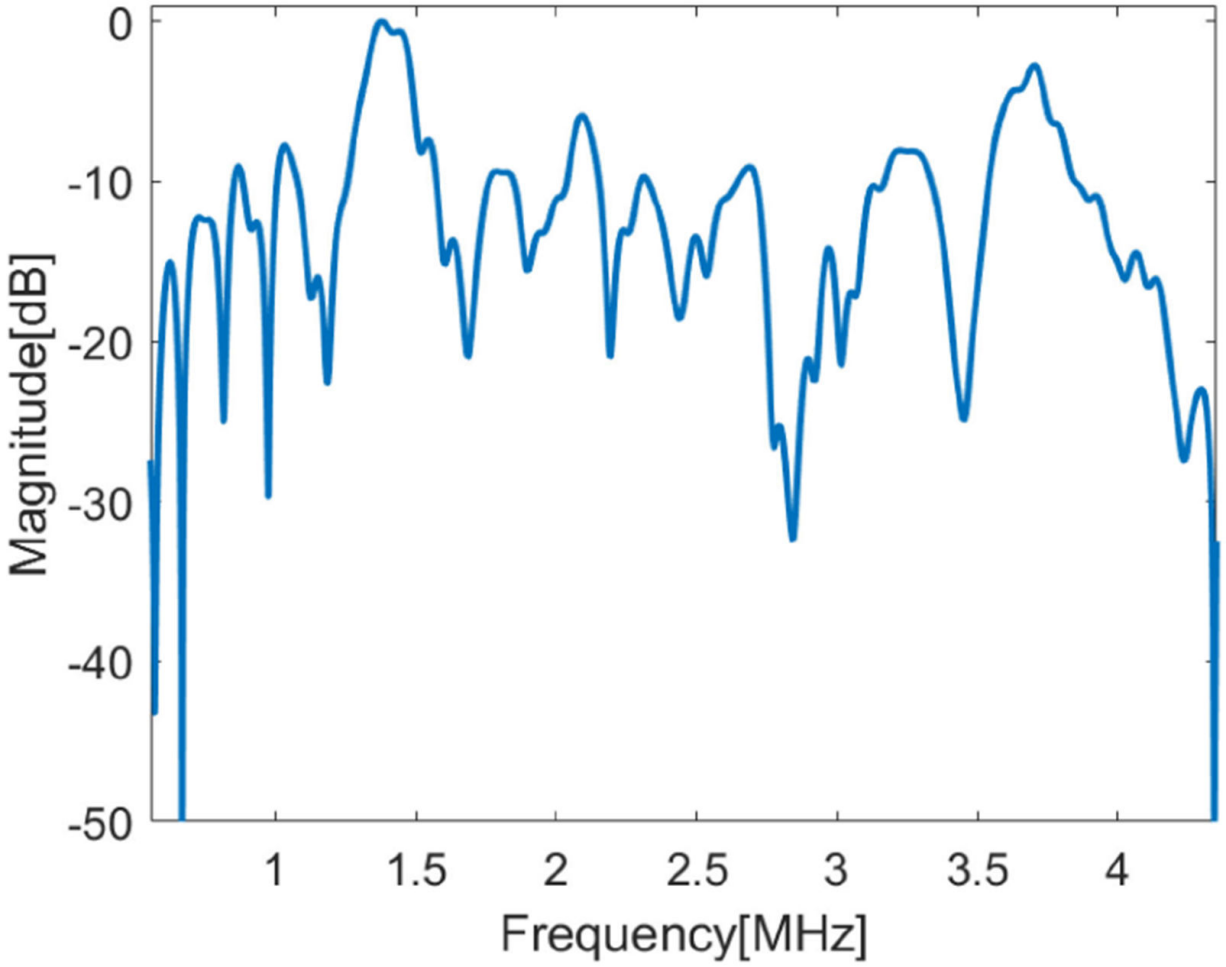
Frequency response of the combined microcrystal and IP103 array averaged over 64 channels (measured in water) for a pitch-catch configuration.

Waveforms corresponding to each frame of data, along with the pilot symbols, were generated via Matlab using custom scripts and the waveforms were loaded into the AWG, which was connected to the microcrystal acting as the transmitter. To use the voltage levels that might be achieved in a capsule endoscopy, we limited the highest transmit voltage to 1 V peak. For the receiver in the phantom experiments we used the IP103 phased linear array and a Verasonics Vantage 128 ultrasound imaging research platform. As the Verasonics system works in the Matlab environment, we could easily retrieve the radio frequency data and perform demodulation in a continuous way. The integrated analog front end of Verasonics provides over 40 dB in gain along with a programmable low pass filter. In this way, there was no need to use an external amplifier to amplify the weak signal from the array elements, which reduced the system complexity. Because the source and receiver were not matched in their frequency bandwidth, we measured the combined frequency response of the microcrystal and IP103 array in the pitch-catch configuration. Fig.8 shows the frequency response of microcrystal and IP103 array measured in water and averaged over 64 channels.

### D. FPGA Implementation

After successfully demonstrating the video streaming with our laboratory system and to move to a smaller hardware footprint, an FPGA board was used for producing the transmission signals. We used a Redpitaya STEM 125-14 as the hardware development platform for the transmitter. The platform uses a Xilinx Zynq 7010 FPGA and dual channel 125 MSPS 14-bit digital to analog converter (DAC) along with an onboard amplification circuit. The FPGA design is comprised of three major parts: camera interface, OFDM modulator and digital up converter. The camera interface was written in Verilog using Xilinx Vivado IDE. The OFDM modulator and digital up converter were designed and generated inside the Xilinx System Generator for digital signal processing (DSP). The modulation scheme described in part B was implemented on the FPGA. The digital up converter design was modified to fit into the limited DSP resources on the FPGA. This design could transmit a 160*50 RGB565 color image from an OV7670 VGA camera module for up to 30 frames per second. The resource utilization and power estimation of FPGA for this task are provided in Table II. The DAC provided two single ended outputs, which were connected to a single microcrystal differentially providing a maximum of 1 Vpp for transmission. We used the FPGA system with the ultrasonic microcrystal for video transmission through the phantom and for the *in vivo* experiments.

### E. In Vivo Setup

The protocol was approved by the Institutional Animal Care and Use Committee (IACUC) at the University of Illinois at Urbana-Champaign. A New Zealand White rabbit was used in the *in vivo* experiments. The rabbit was anesthetized with isoflurane during the procedure and euthanized after the procedure. During anesthesia, the rabbit was placed on its back and its abdomen was shaved and depilated. A small incision was made in the abdomen of the rabbit on its left side below the ribcage. The microcrystal, which was connected via cable to the FPGA, was inserted inside the abdomen approximately 2 cm from the outer skin surface. The FPGA was also connected to a small camera outside of the rabbit. An array probe (C5-2) connected to the Verasonics system was used to receive the signals through the rabbit abdomen. Ultrasonic gel was used to couple the array probe to the outside of the abdomen. Signals were transmitted via ultrasound from within the rabbit abdomen using the embedded microcrystal out to the array sitting on the outer surface a few cm away.

## III. Results

### A. Phantom Results

**Fig. 9.**
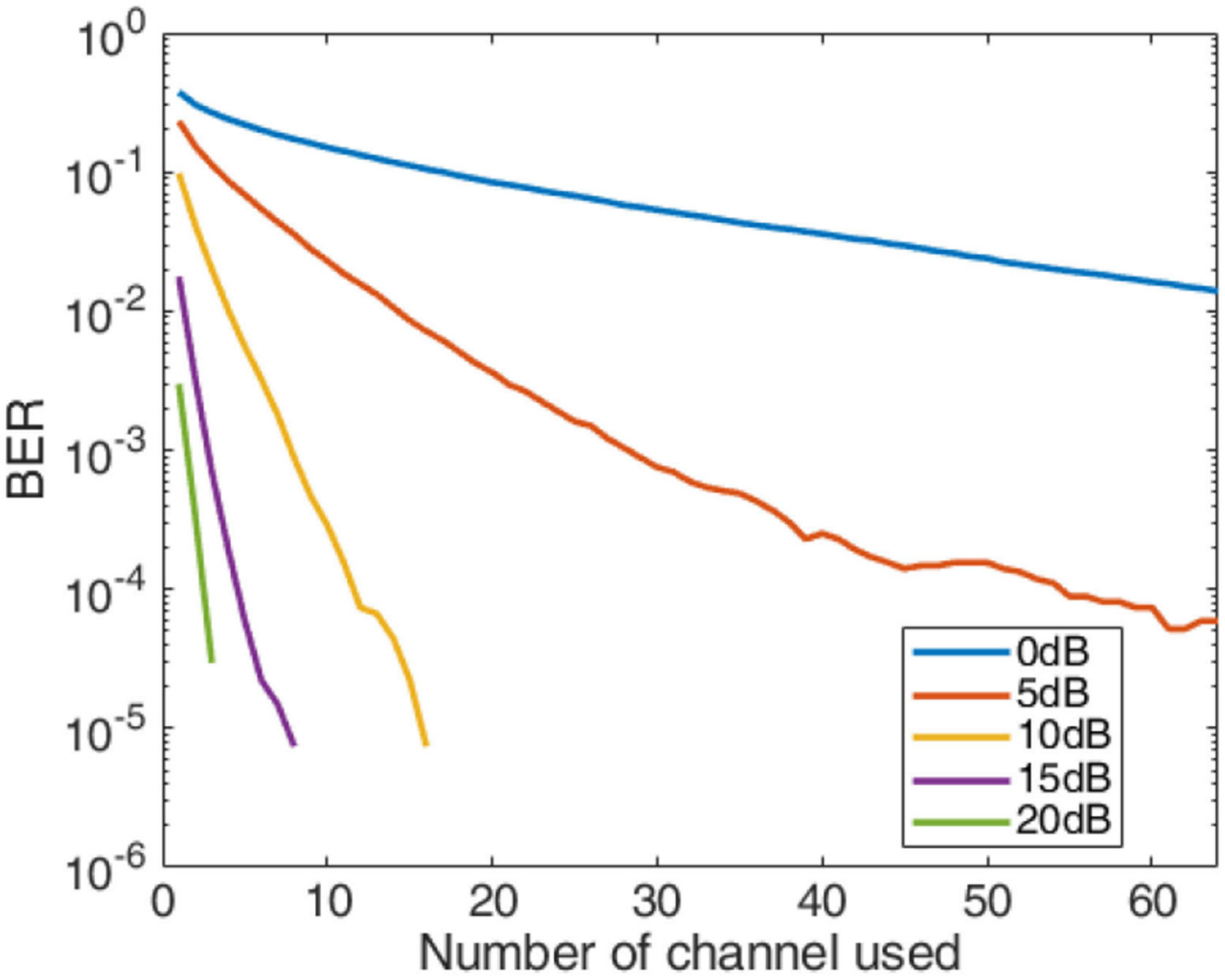
BER results of 16 QAM simulation with measured phantom FIR.

By convolving the FIR measured with IP103’s 64 receiving elements from the phantom with the passband signal, we were able to apply the channel to the up converted signal to perform the simulations over a realistic channel model provided by the phantom. The results are shown in Fig. 9. As Eb/N0, which is defined as the energy per bit over noise power, increased, the MRC results provided better bit-error-rate (BER) performance, which improves as the number of channels increases.

**Fig. 10.**
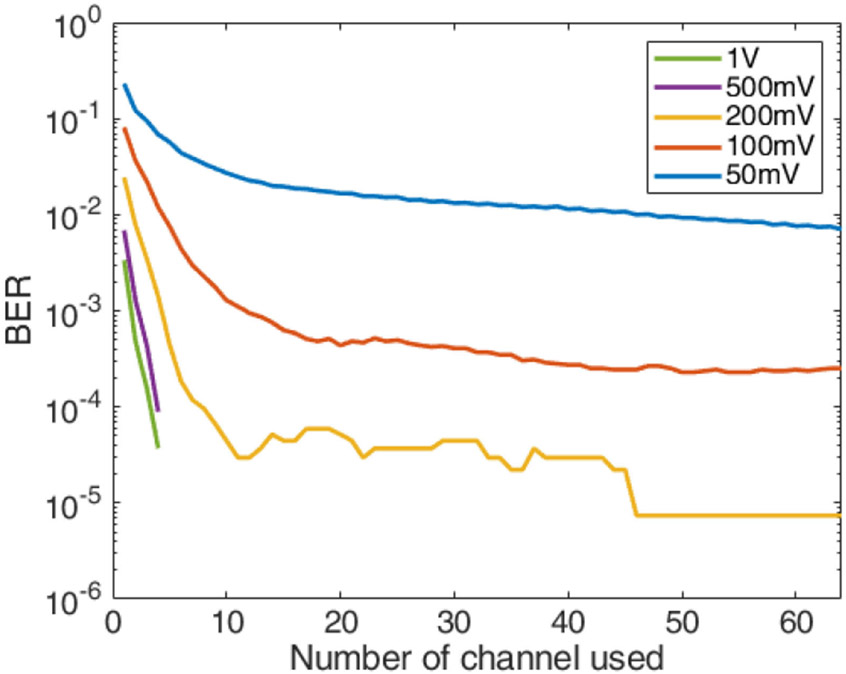
BER performance for 16 QAM through the phantom.

We transmitted the up converted OFDM signal through the phantom and recorded the received signals. By varying the maximum output voltage of the AWG from 50 mV to 1 V peak we achieved both 16 QAM and 256 QAM transmissions with 937.5 KHz bandwidth but with different resulting SNR values. The 16 QAM results are shown in Fig. 10 for different numbers of receive elements. When the maximum transmit voltage was 500 mV and above, there were no errors in received data, which implies that the BER was lower than 7.4e-6 with data rate of 3 Mbps.

**Fig. 11.**
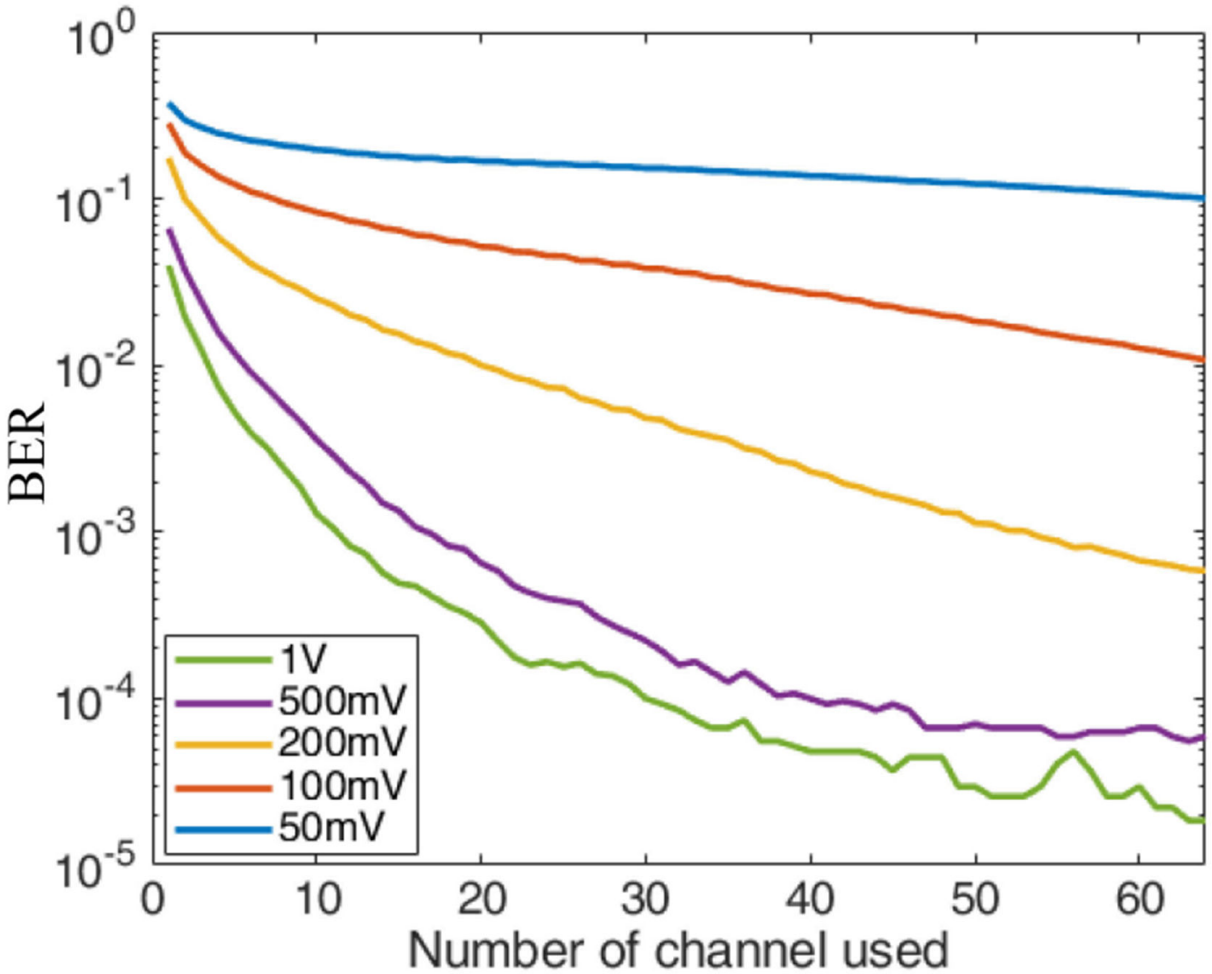
BER performance for 256 QAM with 937.5 KHz bandwidth through the phantom.

The BER performance of 256 QAM is shown in Fig. 11. A BER of 1.8e-5 was achieved when the transmit voltage was 1 V peak with a data rate of 6 Mbps. In terms of BER, the 256 QAM performance was not as good as 16 QAM because we doubled the bits for 256 QAM but the transmit power was unchanged. The Eb/N0 was half of that of 16 QAM. While the BER was not as good as the 16 QAM signals, the BER was still low and acceptable for video streaming with moderate forward error correction.

**Fig. 12.**
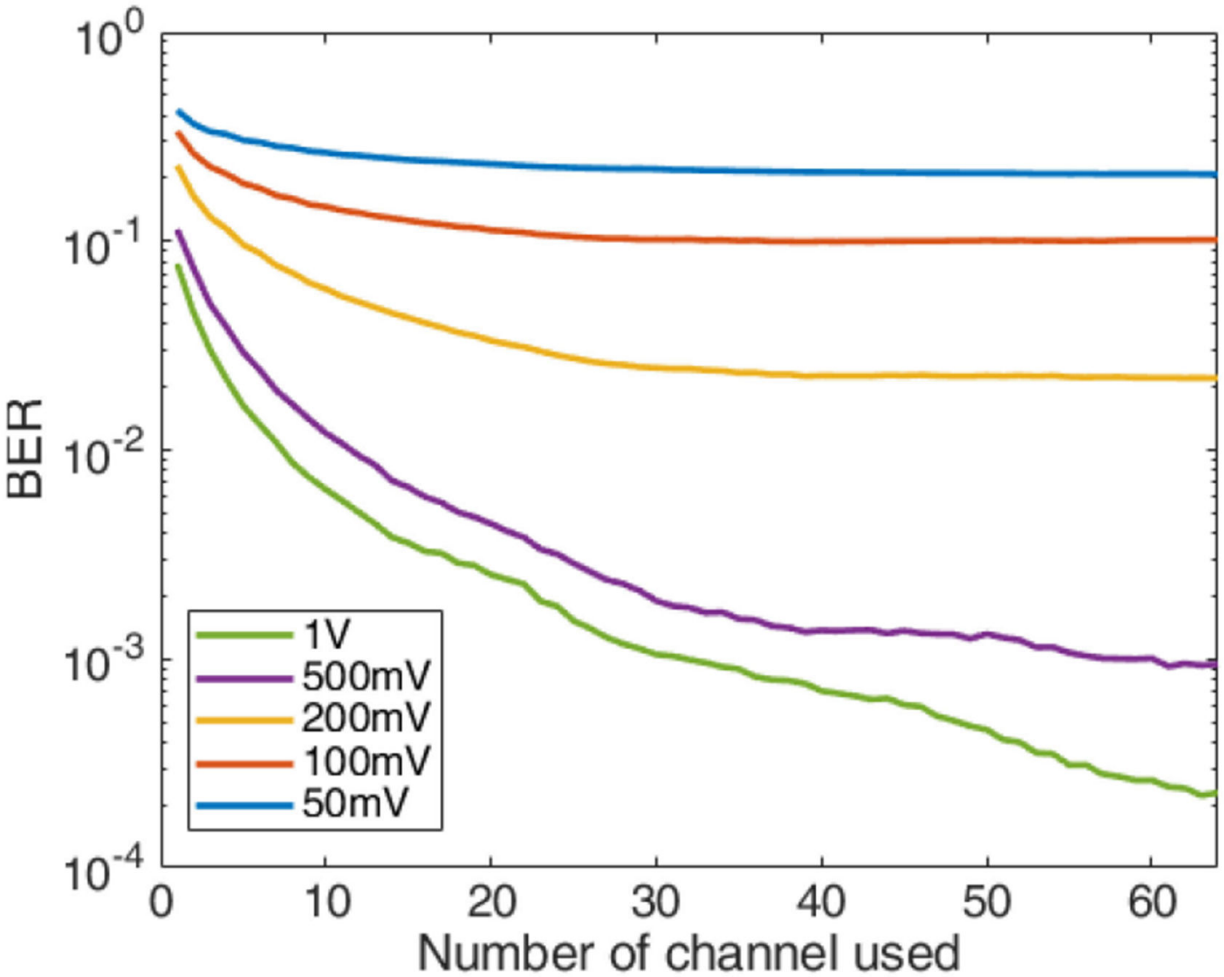
BER performance for 256 QAM with 2.3 MHz bandwidth through the phantom.

The BER performance for the high bandwidth mode, which used a 2.3 MHz bandwidth, is shown in Fig. 12. A BER of 2e-4 was achieved when the transmit voltage was 1 V peak with a corresponding data rate of 15 Mbps.

**Fig. 13.**
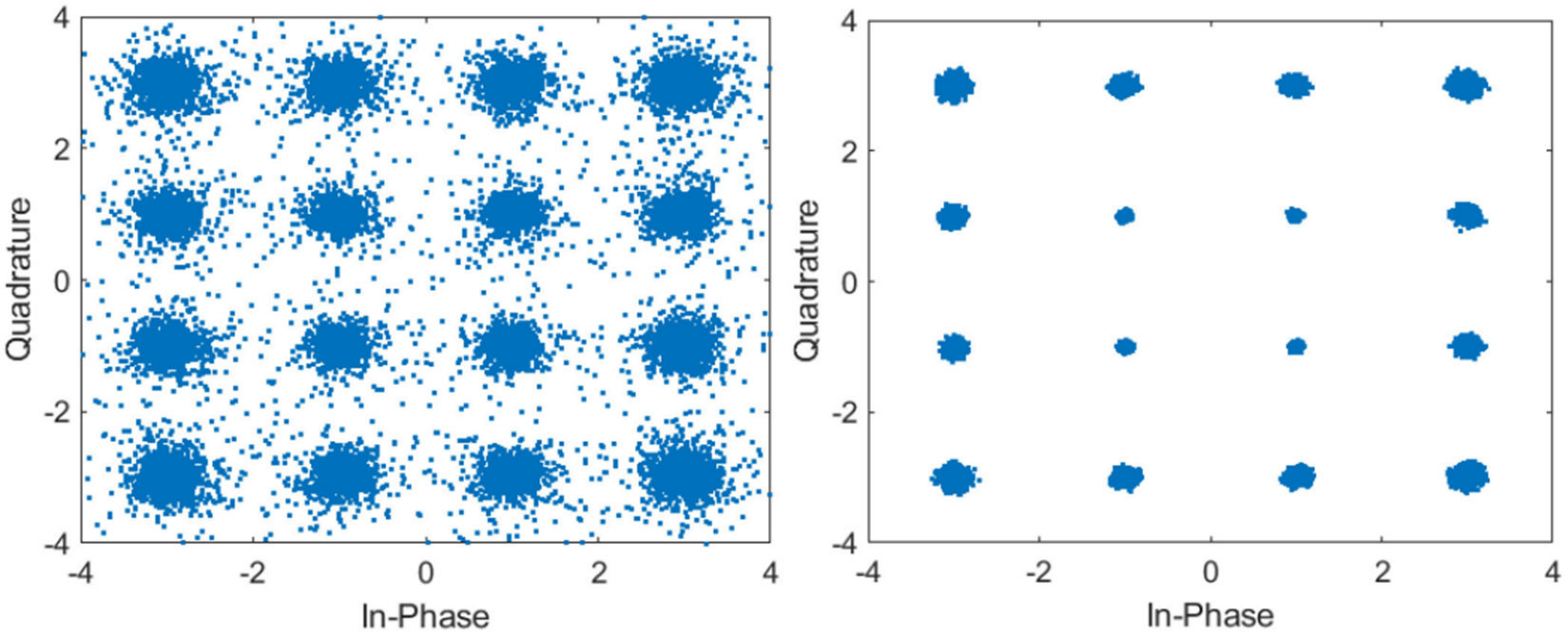
16 QAM constellation diagram when using a single receive channel (left) and using 64 receive channels and MRC (right).

The constellation diagrams of both 16 QAM and 256 QAM at 1 V peak transmit voltage are also presented. Fig. 13 shows the constellation diagrams when using a single channel for receive and 64 channels MRC. In 16 QAM mode a data rate of 3 Mbps was achieved using 64 channels. The average error vector magnitude (EVM) decreased from −20.8 dB to −33.1 dB as more channels were used.

**Fig. 14.**
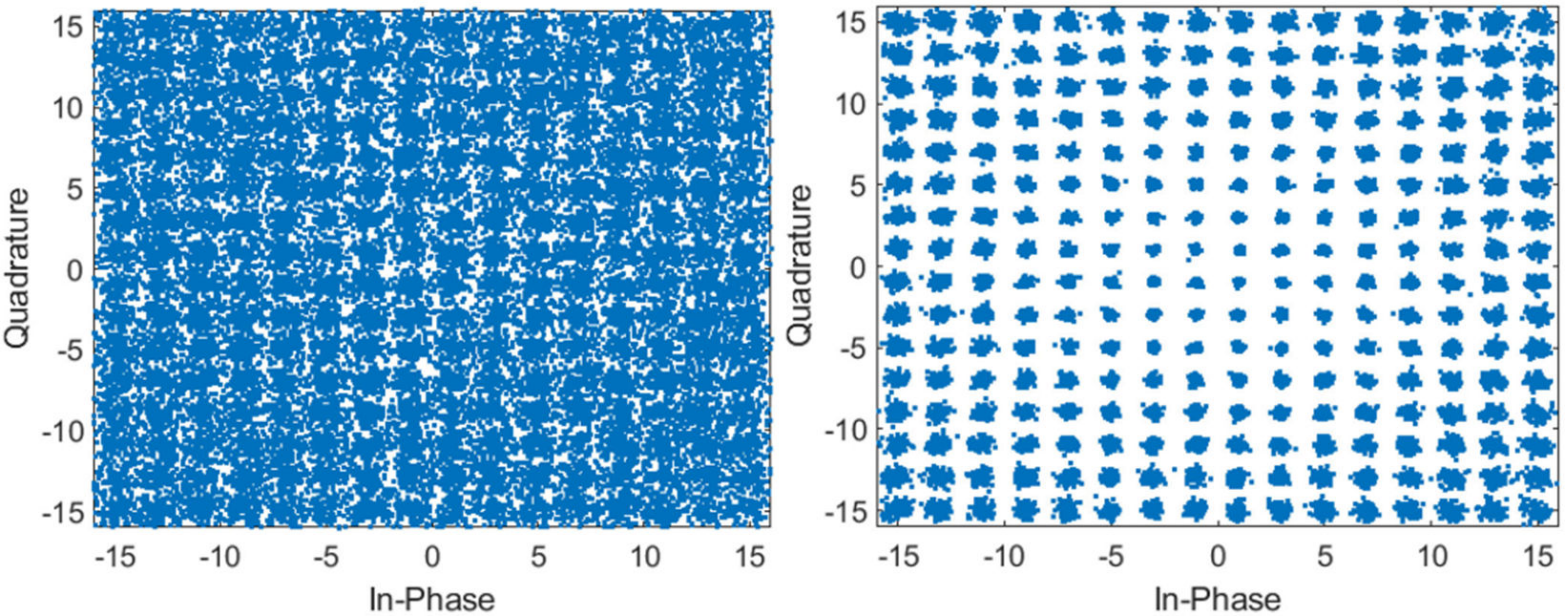
256 QAM constellation diagram when using a single receive channel (left) and using 64 receive channels and MRC (right).

Fig. 14 shows the constellation diagrams for a single receive channel and 64 receive channels using MRC when using 256 QAM. Using 256 QAM mode a data rate of 6 Mbps was achieved. The average EVM decreased from −23 dB to −33.3dB as the channel count increased.

**Fig. 15.**
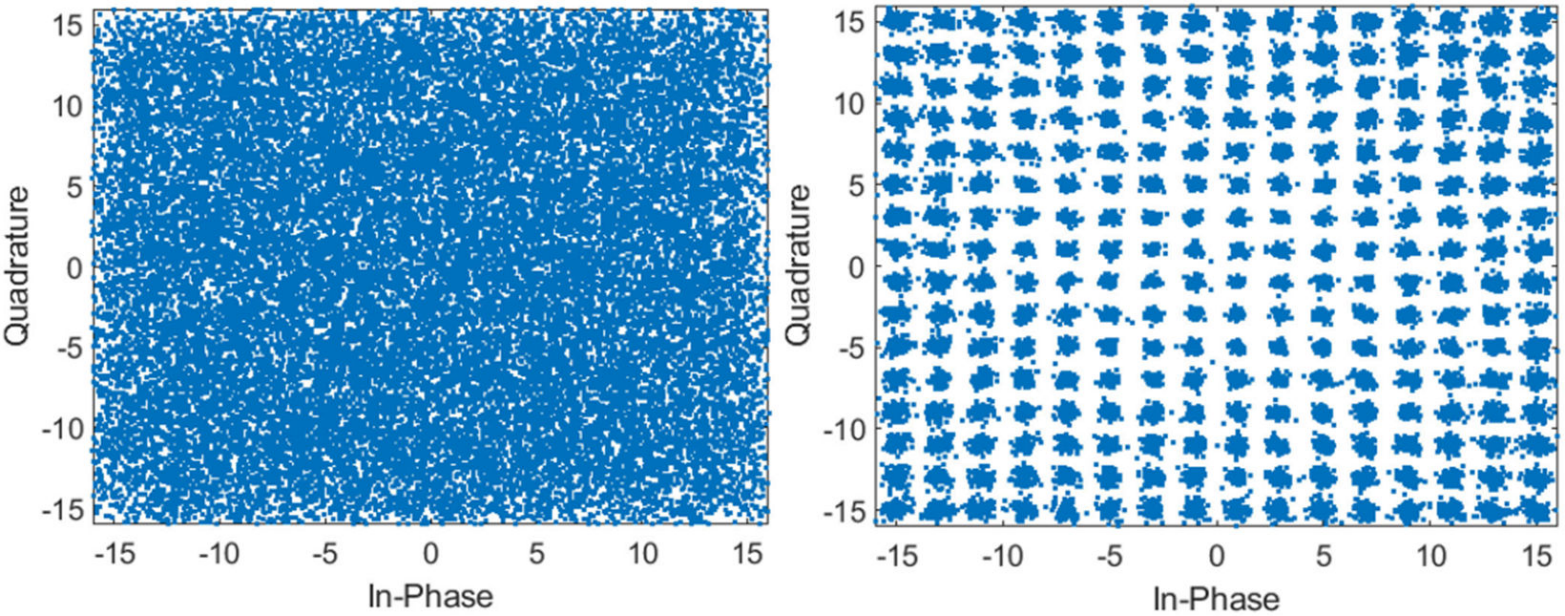
High bandwidth 256 QAM constellation diagram when using a single receive channel (left) and using 64 receive channels and MRC (right).

Fig. 15 shows the constellation diagrams when using a single receive channel and 64 receive channels and MRC in the high bandwidth mode. Using the high bandwidth mode with 256 QAM, a data rate of 15 Mbps was achieved. The average EVM decreased from −17.2 dB to −32.2 dB as the channel count increased to 64 elements.

**Fig. 16.**
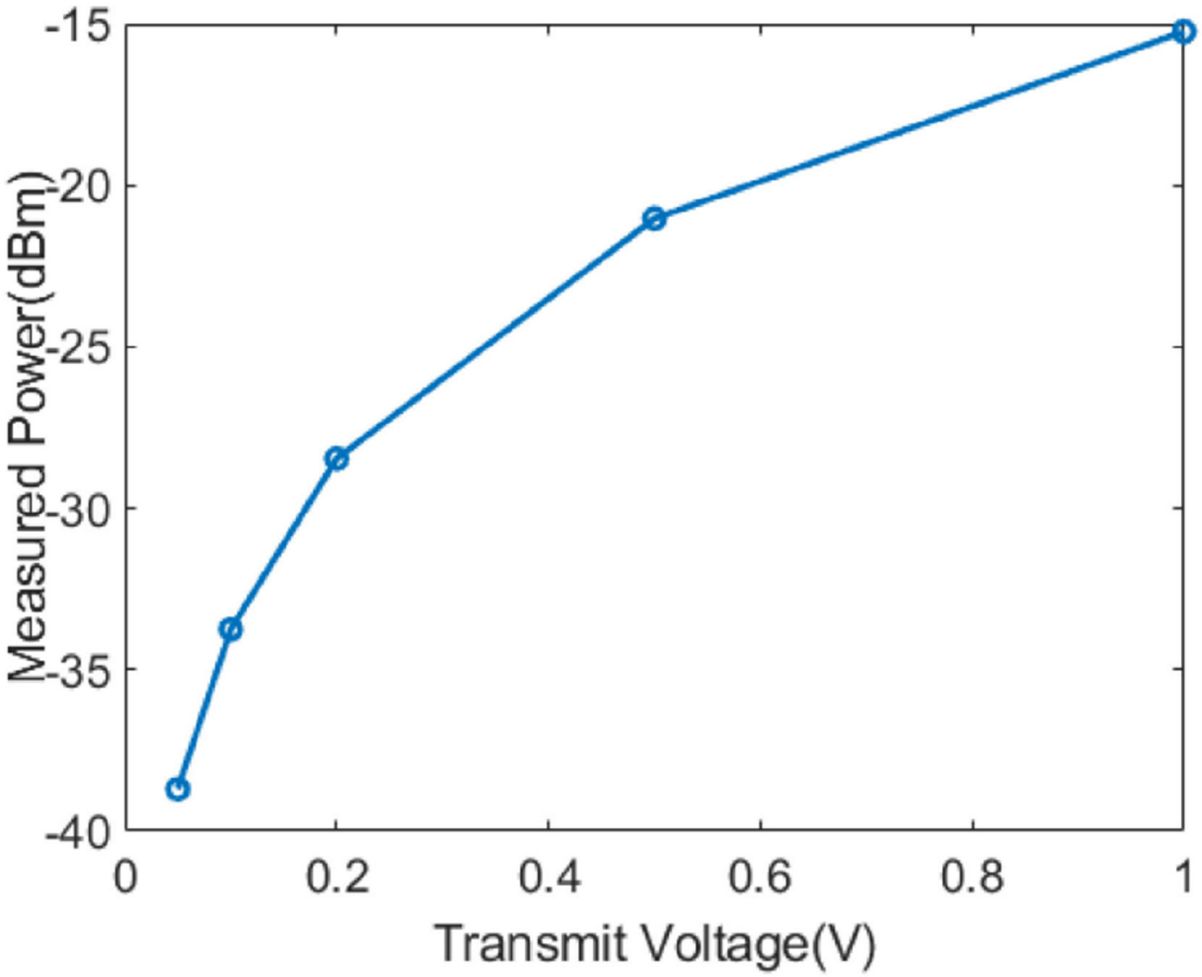
Measured transmit power with hydrophone at different AWG transmit voltage.

The actual transmit pressures at different AWG transmit voltages were measured under water with a hydrophone. The ultrasonic pressures were converted to transmit powers and the results are shown in Fig. 16.

**Fig. 17.**
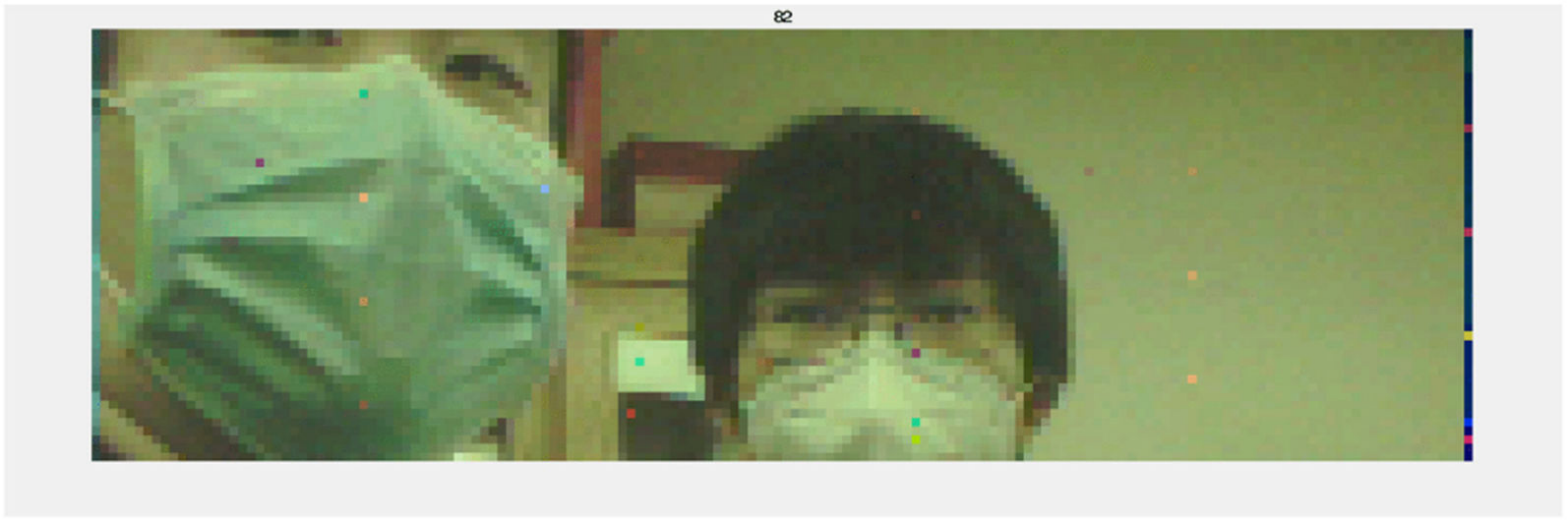
Sample image from video transmitted through the phantom using 16 QAM.

We tested the real video transmission with the FPGA design through the phantom. Fig. 17 shows an image frame received with IP103 probe through the phantom. The image had 160*50 resolution in RGB565 format (16 bits per pixel). The maximum frame rate was 0.5 frame per second when using 10 channels and MRC mode. Performance was limited by the processing speed of host computer. If not limited by the receiver, the FPGA could transmit 24 frames per second, at a 3 Mbps data rate. Due to the limitation of FPGA resources, we did not test the high bandwidth mode with FPGA.

A summary of performance for each operation mode is listed in Table III. Using the array system, we were able to use a small microcrystal with lower power output and still achieve data rates of 15 Mbps through 6.5 cm of tissue-mimicking material.

### B. In Vivo Results

**Fig. 18.**
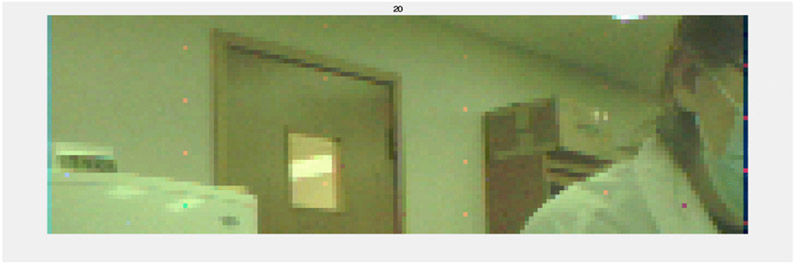
Sample image from video transmitted through rabbit abdomen using 16 QAM.

Fig. 18 shows an image frame received when streaming video signals with ultrasound through the abdomen of a rabbit *in vivo* using a C5-2 curvilinear array probe connected to the Verasonics system. To more easily detect the ultrasonic signal in the animal experiments, we chose to use the C5-2 array probe because it has a wider head laterally and weaker elevational focus compared to the IP103. Therefore, the alignment between the transmitter and array probe did not need to be as exact. The transmitting crystal was embedded at approximately 2 cm inside the rabbit abdomen. The FPGA was used to generate the signals *in vivo* and, therefore, the high bandwidth mode was not tested *in vivo*. Due to the processing speed limitation of the Verasonics system, we set the number of channels used for MRC to 10 for the image transmission at data rate of 3 Mbps. The frame rate and resolution were the same as in the phantom.

## IV. Discussion

In this study, we designed an OFDM modulation scheme, which can use ultrasound as a communication channel to transfer signals at HD video rates. The transmission signals were constructed on an FPGA board to enable real-time video transmission on a small platform. The performance of the communication scheme indicated that the communication protocol was suitable for video capable ultrasound capsule endoscopy. The FPGA transmitted signals at low power while still achieving data rates of up to 15 Mbps.

The use of an array at the receiving end allowed for improved transmission rates over single transducer receivers. Using a single channel on the array resulted in low SNR, which translated to lower data rates to maintain low BER. However, by combining the data from multiple channels of the array, the SNR was increased allowing data rates of up to 15 Mbps with BER of 2e-4. Therefore, the study demonstrates the importance of the receiving hardware in improving signal detection and conversion when using small ultrasonic sources, such as might be used in small implantable medical devices. Furthermore, the array reduced the need to perfectly align the source and receiver. In this case, a 1D array was used allowing focusing and alignment along the lateral dimension.

These high data rates were achieved through a tissue-mimicking phantom, which was constructed to model what might be observed from transmission through the human abdomen. *In vivo* experiments in a rabbit abdomen also demonstrated the ability to transmit video using ultrasound as a communication medium with data rates of 3 Mbps. Table IV provides a comparison of the data rate, BER and system parameters between the setup provided here and the results from other studies. Our results demonstrate high data rates (>3 Mbps) even using unfocused microcrystals as transmitters.

The real-time video transmission based on the current platform is still limited by the processing ability of the software-based receiver. As the active receive channel number increased, the frame rate dropped rapidly. As the number of active receive channels increased, we needed to perform DDC and FFT on every active receiving channel, which means the number of filters and complex multipliers were linearly increasing with the number of active receive channels. This multiple channel calculation can be parallelized for speed up allowing real-time video. For the next step, a hardware based multichannel receiver will be designed that moves all the receive processing, especially DDC and FFT, to an FPGA. Channel coding and video coding will also be implemented to further improve performance.

In this study, small source at 1 MHz that did not have focusing was selected. Higher SNR could be achieved with a focused source. However, a focused beam is highly directional requiring a receiver outside of the body to be aligned with the source. This will add more difficulty in locating a source embedded in the body. The use of a higher frequency source could result in a higher bandwidth and more directionality. However, higher frequencies attenuate more rapidly resulting in a loss of penetration depth. Therefore, the choice of a small 1.2 MHz microcrystal source balanced the need for higher SNR versus directionality.

Furthermore, to move toward a small in-body medical device, like capsule endoscopy, the form factor of the transmit hardware needs to be minimized to fit the size of the device. While camera and ultrasonic transducer options exist that would fit in a small device like an endoscopy capsule, to our knowledge at this time off-the-shelf processing hardware solutions are not available that fit the required footprint. One solution is to design a mixed signal System-on-Chip (SoC) that incorporates video encoding, channel encoding, OFDM modulation and DAC on a single chip. SoC designs and hardware have been constructed for this purpose [20]. The power supply, which is limited by the onboard battery, is also a challenge. A highly integrated SoC can lower the power consumption compared to a separate FPGA and DAC architecture as we currently use allowing for longer operation of an ultrasound-based in-body medical device.

## Figures and Tables

**TABLE I:** Modulation Parameters

Parameter	Symbol	Value	Parameter	Symbol	Value
**FFT size**	*N_FFT_*	4096	Sample Rate	*F_s_*	1.25/3.1 3 MSPS
**Active Carriers**	*N_AC_*	3072	Modulation Depth	*M*	16/256
**Subcarrier Bandwidth**	Δ*_f_*	305/763 Hz	Center Frequency	*F_C_*	2.4/3.2 MHz
**CP length**	*T_CP_*	409/164 μS	Block distance	*D_pilot_*	12

**TABLE II:** FPGA Resource Utilization and Power Consumption

Parameter	Usage	Parameter	Usage
**Look Up Table**	36%	DSP slice	73%
**Flip-Flop**	28%	Dynamic Power	0.264 W
**Block RAM**	73%	Static Power	0.098 W

**TABLE III:** Performance Summary (BER)

Modulation	16 QAM		256 QAM		256 QAM	
Bandwidth	937.5 kHz		937.5 kHz		2.343 MHz	
Data Rate	3 Mbps		6 Mbps		15.2 Mbps	
Voltage	Single	MRC	Single	MRC	Single	MRC
1V	2.619e-3	<7.398e-6	0.099	1.850e-5	0.133	2.027e-4
500 mV	5.526e-3	<7.398e-6	0.066	5.919e-5	0.172	7.287e-4
200 mV	2.421e-2	7.398e-6	0.176	5.845e-4	0.302	1.870e-2
100 mV	8.000e-2	2.959e-5	0.284	0.011	0.391	0.101
50 mV	0.227	3.033e-3	0.376	0.010	0.445	0.220

**TABLE IV:** Performance Comparison

	[1]	[15]	[11]	PillCam[19]	This work
**Medium**	Pork loin	Phantom	beef	Human Body	Phantom
**Transducer**	Focus-Focus	Focus-Focus	Single-Single	RF	Single-Array
**Distance**	5.86 cm	10 cm	10 cm		6.5 cm
**Modulation**	64 QAM	OFDM+8 QAM	OFDM+16 QAM	FSK	OFDM+256 QAM
**Data Rate**	30 Mbps	12 Mbps	340 kbps	800 kbps	6 Mbps/15 Mbps
**BER**	<1e-4	1.9e-4	<1e-4		1.8e-5/2e-4
